# Development and Blind Clinical Validation of a MicroRNA Based Predictor of Response to Treatment with R-CHO(E)P in DLBCL

**DOI:** 10.1371/journal.pone.0115538

**Published:** 2015-02-18

**Authors:** Steen Knudsen, Christoffer Hother, Kirsten Grønbæk, Thomas Jensen, Anker Hansen, Wiktor Mazin, Jesper Dahlgaard, Michael Boe Møller, Elizabeth Ralfkiær, Peter de Nully Brown

**Affiliations:** 1 Medical Prognosis Institute, Hørsholm, Denmark; 2 Rigshospitalet, Department of Hematology, Copenhagen, Denmark; 3 Rigshospitalet, Department of Pathology, Copenhagen, Denmark; 4 Odense University Hospital, Department of Pathology, Odense, Denmark; University of North Carolina at Chapel Hill, UNITED STATES

## Abstract

MicroRNAs (miRNA) are a group of short noncoding RNAs that regulate gene expression at the posttranscriptional level. It has been shown that microRNAs are independent predictors of outcome in patients with diffuse large B-cell lymphoma (DLBCL) treated with the drug combination R-CHOP. Based on the measured growth inhibition of 60 human cancer cell lines (NCI60) in the presence of doxorubicine, cyclophosphamide, vincristine and etoposide as well as the baseline microRNA expression of the 60 cell lines, a microRNA based response predictor to CHOP was developed. The response predictor consisting of 20 microRNAs was blindly validated in a cohort of 116 de novo DLBCL patients treated with R-CHOP or R-CHOEP as first line treatment. The predicted sensitivity based on diagnostic FFPE samples matched the clinical response, with decreasing sensitivity in poor responders (P = 0.03). When the International Prognostic Index (IPI) was included in the prediction analysis, the separation between responders and non-responders improved (P = 0.001). Thirteen patients developed relapse, and five patients predicted sensitive to their second and third line treatment survived a median 1194 days, while eight patients predicted not sensitive to their second and third line treatment survived a median 187 days (90% CI: 485 days versus 227 days). Among the latter group it was predicted that four would have been sensitive to another second line treatment than the one they received. The predictions were almost the same when diagnostic biopsies were used as when relapse biopsies were used. These preliminary findings warrant testing in a larger cohort of relapse patients to confirm whether the miRNA based predictor can select the optimal second line treatment and increase survival.

## Introduction

Diffuse large B-cell lymphoma (DLBCL) account for 40% of all adult non-Hodgkin lymphomas. It is characterized by a marked biological heterogeneity and variable clinical presentation and clinical course. Although modern therapy has increased survival substantially with 5 year survival rates more than 60%, it is necessary to identify biologic predictive markers that can predict response to specific treatment regimens which is the key issue in personalized medicine.

The standard R-CHOP first line treatment is highly effective. More than 80% respond to this treatment. Patients that suffer a relapse after first line treatment have a substantially worse prognosis, however. If it were possible to identify the optimal second line treatment for each individual in this group, the prognosis may improve.

DLBCL represents one of the early successes for mRNA-based microarrays. Alizadeh et al [1] discovered two novel subtypes: Germinal Centre B-like (GCB), and Activated B-like (ABC) that had different prognostic characteristics. This was followed by other gene expression signatures with prognostic relevance for DLBCL [2, 3]. Also microRNA signatures have been identified that showed similar subgrouping or prognostic relevance [4–6]. These signatures have not yet entered clinical practice, however.

So far, signatures predictive of specific treatments or treatment alternatives have not been tested in DLBCL.

Numerous papers have addressed the development of predictors based on mRNA isolated from fresh frozen patient samples. For such predictors to gain wide clinical use, however, it is important that they readily can be implemented in standard practice. For that reason, platforms that readily analyze mRNA or microRNA from formalin-fixed paraffin embedded (FFPE) samples should be used. We have developed predictors based on highly stable microRNA which allows reliable utilization of FFPE samples, that are routinely prepared and stored for most patients in pathology labs thus, allowing for a practical implementation biomarkers and predictors in the clinic.

We have characterized the microRNA transcriptome in the panel of cell lines from NCI60 [7]. The same panel has been tested for sensitivity to a large number of chemotherapy drugs at the NCI. This has allowed us to develop two microRNA profiles, the expression level of which are correlated to the sensitivity to the combination treatments CHOP and CHOEP, respectively, the most widely used standard treatment of DLBCL. It has also allowed us to develop microRNA profiles for all treatments used in second line after relapse.

For the first time we describe the blind clinical validation of such predictive biomarkers in samples from DLBCL patients.

## Methods

### Patients

A total of 130 patients diagnosed with de novo DLBCL treated with R-CHOP or R-CHOEP between 2002 and 2010 with available tissue from a local biobank at Rigshospitalet were identified for the study. From formalin-fixed paraffin embedded (FFPE) tissue samples between 3 and 5 slices of thickness 15 μm were used. Tumor cell content was estimated based on HE staining of an adjacent slice and ranged from 5% to 90%. 116 biopsies yielded RNA of sufficient quantity (at least 400 ng total RNA as quantified by Nanodrop) for further analysis. In addition, we obtained duplicate biopsies from two patients, and relapse biopsies from 10 patients. Clinical data were obtained from the Danish Lymphoma Registry (LYFO) and from patient files. Clinical response was determined as defined by Cheson et al [8]: CR was defined as normal biochemistry, lymph nodes and bone marrow. CRu was defined as CR but with residual tumor above 1.5 cm which has been reduced more than 75% and bone marrow with increased number and size of lymphoid aggregates but without cytological or histological abnormality. PR was defined as 50% reduction in lymph node size and no new lesions. PD was defined as new lesions and more than 25% progression of lymph node size. SD was defined as no progression and unchanged status. Relapse was defined as a new lesion or more than 50% increase in any previously identified node.

### Ethics Statements

This project was approved by the Regional Ethics Committee for the Capital Region of Denmark with approval number H-KF-284246. The approval specifically waived informed consent on the condition of anonymity and on the condition that subjects who have requested no use of their diagnostic samples for research purposes were excluded in agreement with Danish ethical regulations. Patient samples and medical records and information were obtained in an anonymized and/or de-identified form.

### Microarray analysis

MicroRNA was extracted from FFPE using RecoverAll (Ambion, Inc 2130 Woodward St. Austin, TX). MicroRNA was labeled using *FlashTag HSR Biotin RNA Labeling Kit* (Genisphere, PA) and analyzed using GeneChip miRNA version 1.0 arrays (Affymetrix, CA). The resulting raw microRNA data files have been deposited at GEO under accession number GSE40239.

### Predictor Development based on in vitro assay

The growth inhibition (GI50) vectors of 60 cell lines subjected to cyclophosphamide, vincristine or doxorubicin was downloaded from the DTP web site (http://dtp.nci.nih.gov). The -log(GI50) vectors for each of the three drugs were summed before correlating to microRNA expression levels measured using Affymetrix microRNA v. 1.0 on Genisphere FlashTag HSR labeling of Ambion Recoverall isolated microRNA from same cells [7]. 20 microRNAs with a Pearson correlation above 0.25 were considered biomarkers of sensitivity and retained as a response profile for CHOP, as previously described for mRNA-based biomarkers [9, 10]. The entire procedure was repeated for CHOEP, yielding a 30 microRNA response profile. The same procedure was applied for other treatments used in second and third line.

### Prediction of CHOP and CHOEP sensitivity in clinical samples

After RMA normalization of array data from clinical samples, the expression of each microRNA in the response profile was used to predict sensitivity: *Prediction score = mean(microRNAs)* That means that each microRNA in the profile is given equal weight. Next, the prediction score was normalized to a scale from 0 to 100 by a linear transformation of the prediction score of all patient samples. The same procedure was used for the CHOP profile and the CHOEP profile, so we could apply the normalized CHOP prediction score for patients that were treated with CHOP and the normalized CHOEP prediction score for patients that were treated with CHOEP. For the GCB/ABC profile the mean of the ABC subtype microRNAs was subtracted from the mean of the GBC subtype microRNAs. All profiles used are listed in S1 Table.

### Statistical Analysis

The statistical analysis was performed according to a Statistical Analysis Plan (S1 File), with pre-specified success criteria, that was agreed upon before unblinding of the clinical data.

Clinical covariates specified in the statistical analysis plan were combined with the prediction score by giving equal weight to the prediction score and the clinical covariates available by multiplying IPI with 25, which makes the range (125), comparable to that of the Prediction score (100): Combination score = Prediction score—25 * IPI score

This formula was tested on published dataset of mRNA from CHOP treated DLBCL patients [11]. This allowed us to perform power calculations for the design of the microRNA trial: at least 23 responders and 23 non-responders would be required to obtain a power of 0.9 with the Combination score. Although we did not reach the required number of primary non-responders, we decided to unblind the dataset anyway. The primary analysis was a one-sided Wilcoxon test for difference in Combination score between responders (CR+CRu) and non-responders. Overall survival (OS) was defined as the time from first lymphoma diagnosis till death of any cause. Patients still alive at the time of analysis were censored at the last date of data merging between LYFO and the National Central Person Registry. Progression free survival was defined as the time from the first lymphoma diagnosis until lymphoma progression or death as a result of any cause. A log-rank test of survival for patients with a prediction above and below cutoff 50 (on a scale of 0 to 100) was used

Response to relapse treatment was predicted as shown for Combination score above, where the prediction score was the average of second and third line treatments. Relapse patients were divided into predicted sensitive and predicted resistant using a cutoff that was optimized to separate the two groups.

## Results

The demographics of the 116 patients is shown in Table 1.

**Table 1:** Patient demographics.

Variable	Values	N
**Sex**		
	Male	54
	Female	62
**Age**		
	Minimum	22
	Median	63
	Maximum	86
**IPI**		
	0	10
	1	28
	2	37
	3	18
	4	16
	5	7
**Treatment**		
	R-CHOP	95
	R-CHOEP	21
**Survival**		
	Dead	22
	Alive	94
		
**Median observation**		
	Days	1274
		
**Treatment response**		
	CR	57
	CRu	42
	PR	5
	PD	2
	Dead	5
	Unevaluable	5
		
**Relapse**		
	Relapse	13
	No relapse*	98

* excludes patients dead before response evaluation

**Fig 1:**
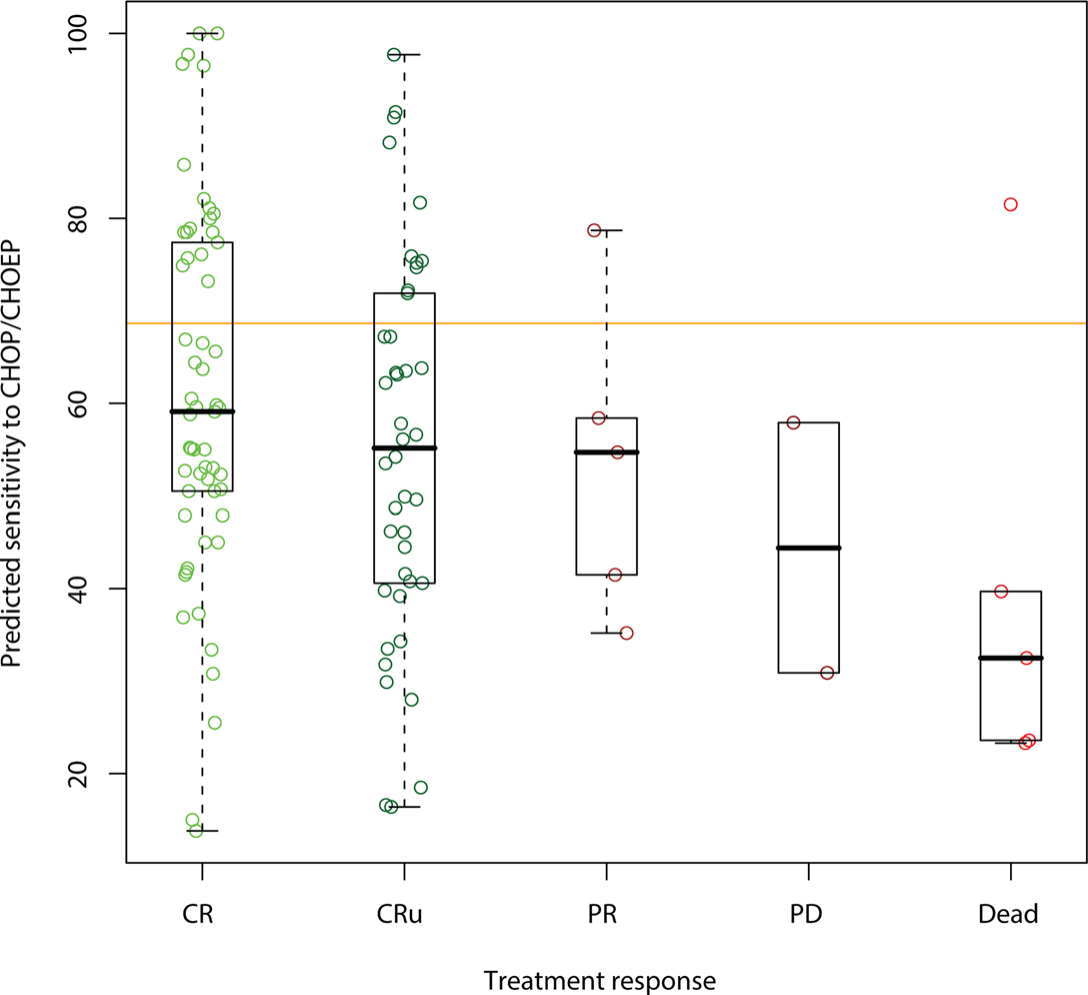
Correlation between predicted sensitivity to CHOP/CHOEP and response to treatment (CC = 0.24, P = 0.006). CR = Complete Remission, CRu = Complete Remission unconfirmed, PR = Partial Remission, PD = Progressive Disease, Dead = dead before response evaluation. A Wilcoxon rank test comparing CR to all other responses gives a p-value of 0.03. The pre-specified cutoff is shown with an orange line. The patient in the last column with a high predicted sensitivity (prediction score 82) died from a relapse within 148 days of diagnosis.

The sensitivity of the 60 cell lines from NCI60 to doxorubicin, vincristine and cyclophosphamide was correlated to the measured base-line concentration of 1756 human microRNAs measured in the same cell lines using Affymetrix GeneChips. We identified 20 microRNAs (Table 2) that, on average, are expressed higher in cell lines sensitive to doxorubicin, vincristine and cyclophosphamide. The correlation of each microRNA to sensitivity in vitro and in vivo is shown in Table 2. It is evident that vincristine and doxorubicin are the main contributors to the 20-microRNA profile. The 20 microRNA expression levels can be turned into a single sensitivity score by taking the average of all 20 microRNAs. This score can be used to predict the sensitivity of a patient to treatment with CHOP by measuring the 20 microRNAs in a tumor biopsy sample (FFPE). When we turned their expression level into a prediction score on a scale from zero to 100 in a cohort of 116 DLBCL patients, the score correlated with the response to treatment with CHOP (Fig. 1).

**Table 2:** List of 20 microRNAs and snoRNAs predictive of sensitivity to CHOP.

microRNA	Vin	Dox	Cyclo	Remission	P-value
ACA48_x_st	0.22	0.4	0.0013	-0.091	0.83
U55_x_st	0.19	0.38	0.14	-0.14	0.94
hsa-miR-106b-star_st	0.35	0.36	-0.1	0.21	0.012
hsa-miR-106b_st	0.19	0.36	-0.0017	0.077	0.21
hsa-miR-1181_st	0.25	0.33	0.14	-0.057	0.73
hsa-miR-124_st	0.23	0.34	0.094	0.053	0.28
hsa-miR-1299_st	0.19	0.42	0.095	0.069	0.23
hsa-miR-25-star_st	0.32	0.26	-0.14	0.088	0.17
hsa-miR-33b-star_st	0.28	0.33	-0.098	-0.0035	0.51
hsa-miR-432_st	0.29	0.26	0.02	0.27	0.002
hsa-miR-551b-star_st	0.28	0.19	-0.1	0.21	0.013
hsa-miR-629-star_st	0.21	0.4	-0.013	0.15	0.056
hsa-miR-629_st	0.19	0.3	0.013	0.14	0.065
hsa-miR-652_st	0.25	0.33	0.075	0.18	0.026
hsa-miR-654-3p_st	0.19	0.28	0.071	-0.06	0.74
hsa-miR-671-5p_st	0.25	0.36	0.085	-0.034	0.64
hsa-miR-766_st	0.21	0.32	0.084	-0.11	0.88
hsa-miR-877-star_st	0.31	0.42	0.089	-0.18	0.97
hsa-miR-93-star_st	0.3	0.28	-0.1	0.043	0.32
hsa-miR-93_st	0.23	0.35	-0.023	0.081	0.19

The first two RNAs are also known as SNORA48 and SNORD55. hsa-miR-25-star_st is known to regulate TP53 negatively. The miR-106b-25 cluster (miR-106b, miR-93, and miR-25) is involved in E2F1 posttranscriptional regulation and Targets PTEN. hsa-miR-124_st is known to regulate CDK6 and ITGB1. For each RNA, the correlation to in vitro sensitivity to vincristine, doxorubicin and cyclophosphamide is shown together with the correlation to in vivo remission and p-value of correlation to remission.

The International Prognostic Index (IPI) [12] is standardly used for DLBCL patients. For this reason it was essential to evaluate if our prediction score added to the predictive (clinical response) or prognostic (survival) performance of the IPI. Table 3 shows that the combination of prediction score and IPI is superior to either on their own in predicting the primary endpoint, remission. IPI is superior at predicting survival, as this is what it was developed for. The prediction score contributes little to the prediction of these endpoints.

**Table 3:** Summary statistics.

Clinical endpoint	Prediction	IPI	Combined	GCB/ABC
Remission	0.03*	0.06	0.001*	0.06
Overall survival	0.2	0.02*	0.03*	0.03*
Progression free survival	0.4	0.03*	0.02*	0.03*

P-values in Wilcoxon rank tests or logrank test of predictive (Prediction score) and prognostic biomarkers (IPI, GCB/ABC) evaluated for different clinical endpoints (Remission = CR+CRu). Combined is the combination of IPI and Prediction score.

* indicates statistically significant.

Table 4 shows a multivariate analysis of the contributions of IPI and prediction score to the primary endpoint, remission: CR ~ A * Prediction + B * IPI

**Table 4:** Multivariate analysis of the ability of the IPI and Prediction scores to predict remission.

	Estimates for A, B, C	Univariate P	Multivariate P
Prediction score	0.02	0.03*	0.01*
IPI	-0.4	0.01*	0.01*
GCB/ABC	0.01	0.08	0.25

Complete remission (CR) is predicted according to the following formula:: CR ~ A * Prediction + B * IPI + C * GCB/ABC. The best estimate for A and B have a ratio of 18, which is a lower weight for IPI than the 25 we have used in Table 3. The multivariate P-values (one sided) show the contribution of prediction and IPI in a combined prediction. For comparison, the univariate P value of prediction score alone or IPI alone is shown. GCB/ABC does not contribute to this endpoint.

It is seen from Table 4 that the prediction score is an independent and statistically significant contributor of the prediction of the primary endpoint, remission.

Wright et al [13] have published a gene expression profile that divides DLBCL into germinal center B cell-like (GCB) and activated B cell like (ABC). The subgroups have different 5 year survival. The profile has been translated to a microRNA profile using samples for which both gene expression and microRNA profiling is available. Among two published GCB/ABC profiles of 10 microRNAs [14] and 8 microRNAs [6] there were only 2 microRNAs overlapping. When applied to our cohort, the 10-microRNA profile performed better and is reported in Tables 3 and 4. It confirms that the 10 microRNA profile is prognostic but not predictive as our 20 microRNA profile, which does not overlap either of the GCB/ABC signatures.

### Effect of varying cutoff

**Fig 2:**
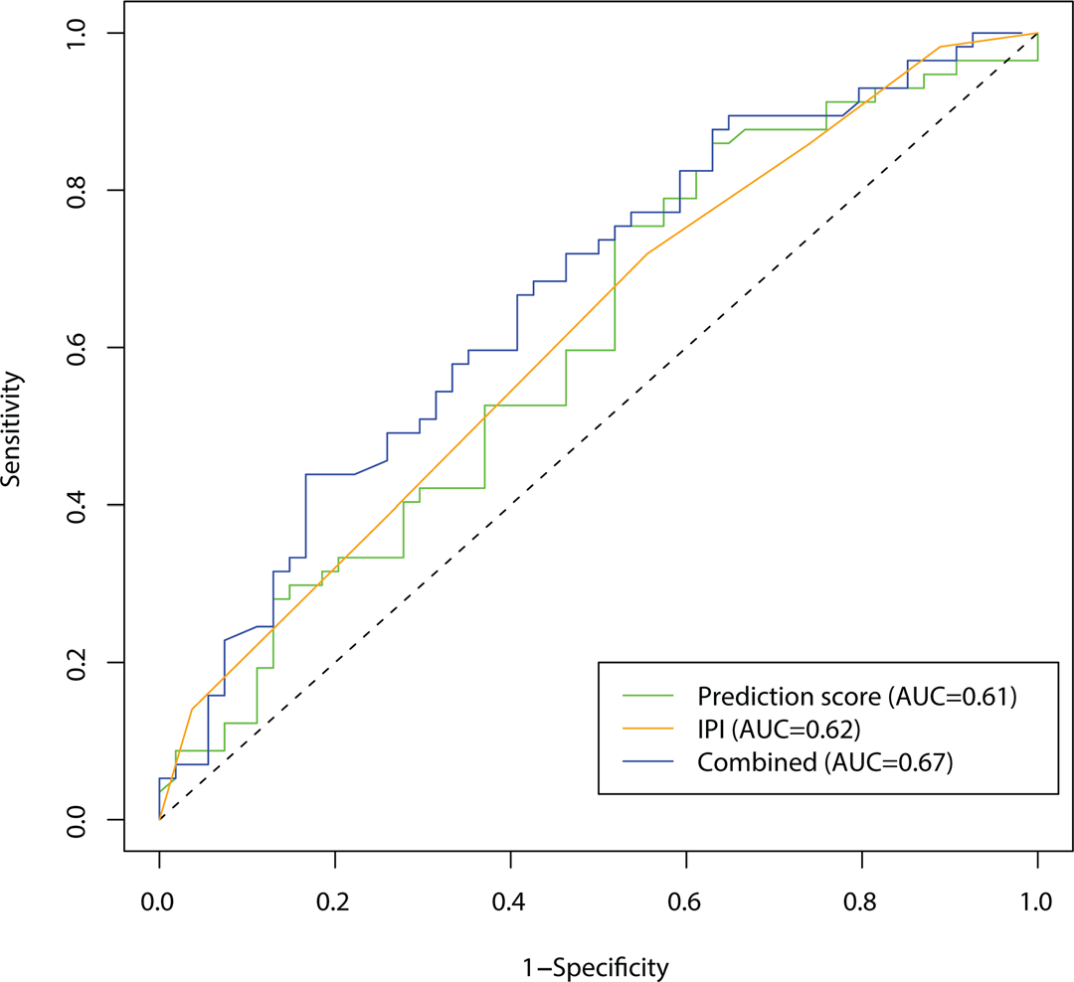
A Receiver Operating Characteristic showing the effect of varying the cutoff. Three measures are compared: Prediction score (green), IPI (orange) and Combined score (blue). By comparing the areas under the curve (AUC) it is seen that the Combined score is superior.

For the statistical analysis, we used a cutoff of 50. Fig. 2 shows the effect of varying this cutoff in a Receiver Operating Characteristic. This allows comparison between different biomarkers and shows that the combination of the prediction score and the IPI score is superior, because it has the largest area under the curve (AUC).

### Relapse treatment

**Fig 3:**
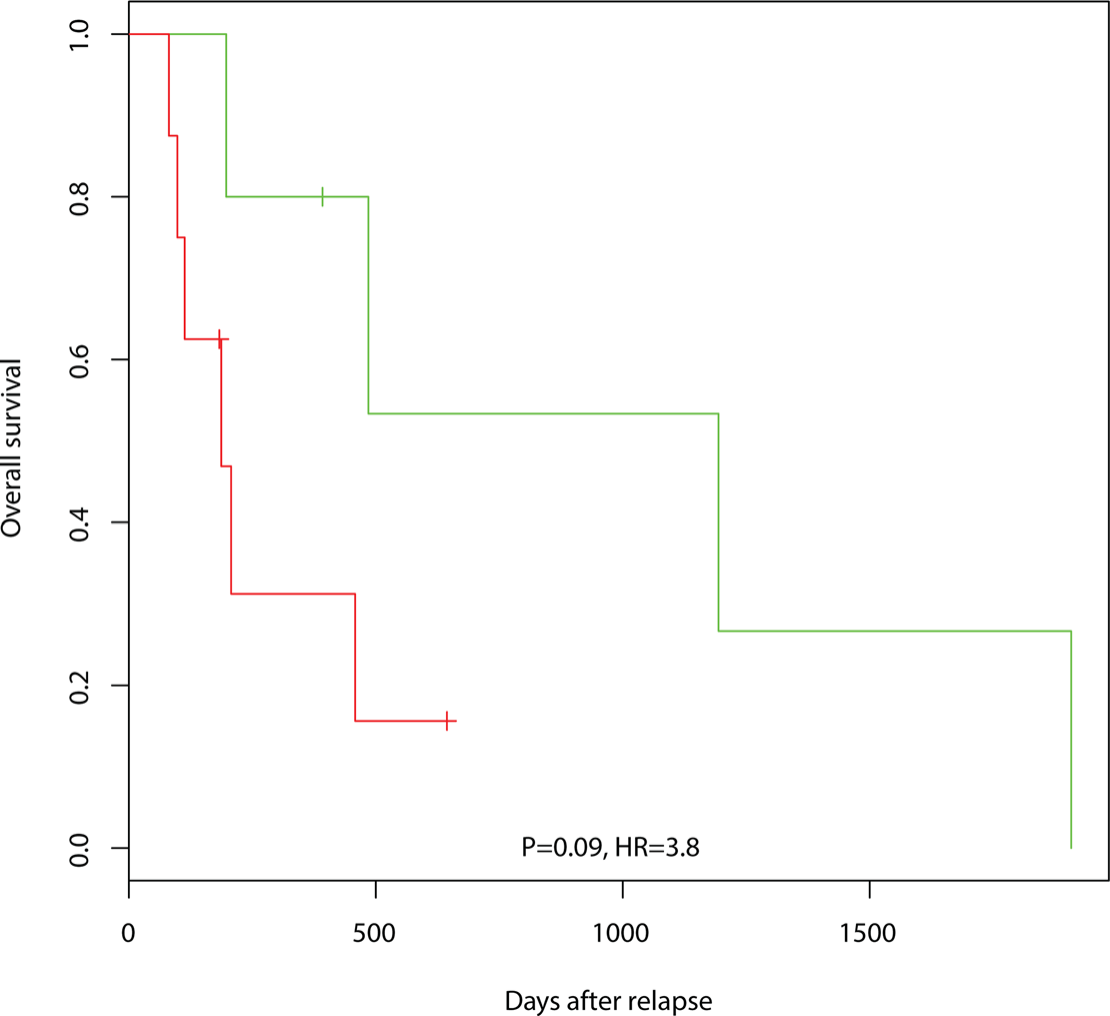
Overall survival after second and third line therapy among thirteen patients with disease relapse. The green line shows the Kaplan-Meier curve of five patients that are predicted sensitive to second and third line treatment received and the red line shows the eight patients predicted resistant.

Thirteen patients had a relapse and were given relapse treatment. The second and third line treatments consisted of the combinations COPE, DHAP, CVP, HDMTX, or MVBCNS. When we predicted the sensitivity of the patients to second and third line treatment, the five patients predicted sensitive to the treatment they received, survived longer (median 1194 days, 90% CI: 485 to >1194 days) than the eight patients that were predicted not sensitive to the treatment received (median 187 days, 90% CI: 98–227 days). The survival of the two groups is shown in Fig. 3, which is based on relapse biopsies available for ten patients, while diagnostic biopsies were used for three patients. If diagnostic biopsies were used from all thirteen patients, the results changed a little, but retained the overall survival characteristics (median 1194 days versus 187 days) if the cutoff between sensitive and resistant was adjusted to obtain the same number of predicted sensitive patients. Among the eight patients that were predicted not sensitive to the treatment they received, it was predicted that three would have been sensitive to ICE and one would have been sensitive to bendamustine.

### Comparison of relapse and diagnostic biopsies

**Fig 4:**
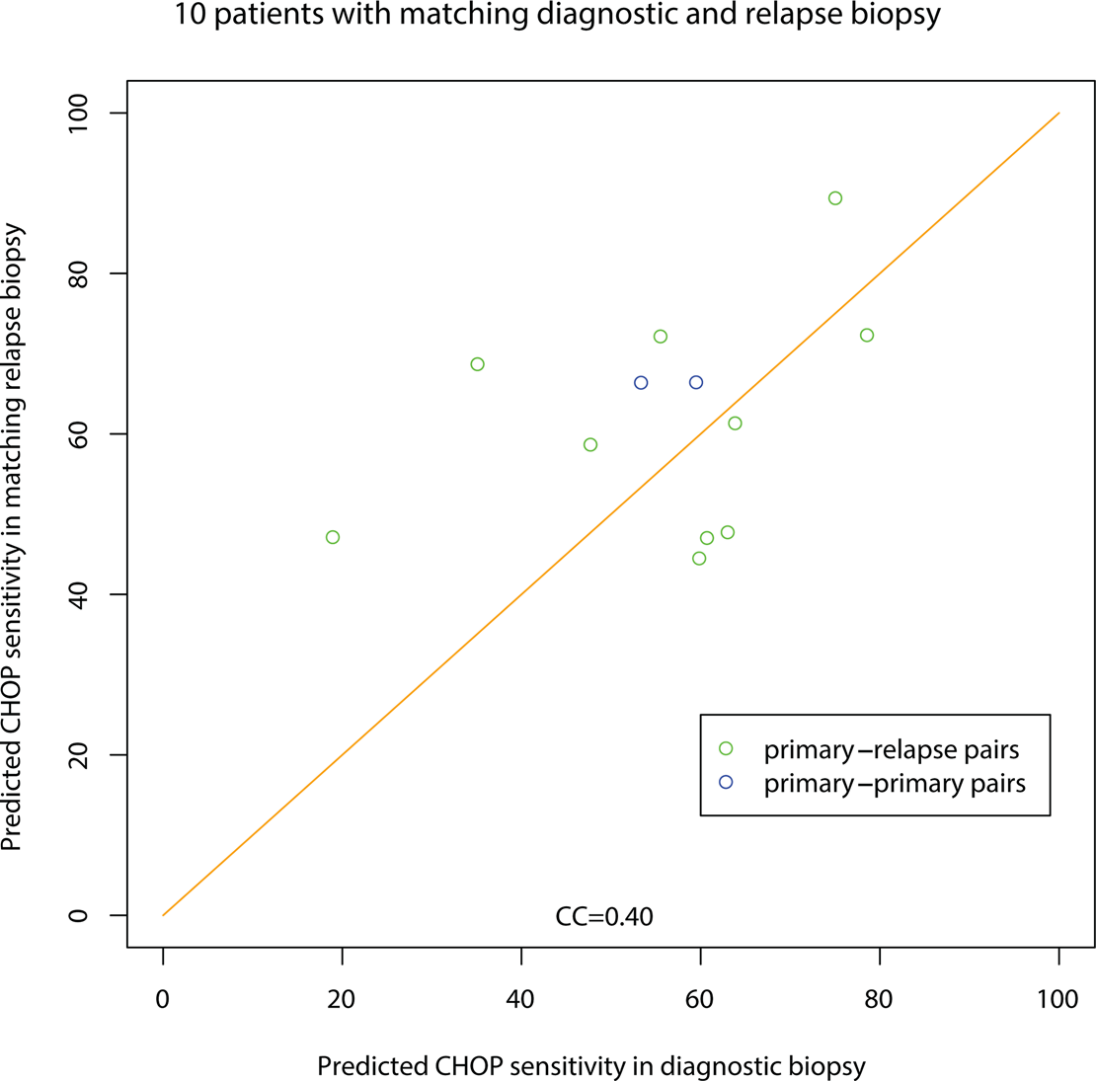
Comparing diagnostic and relapse biopsies. Matching primary-relapse biopsy pairs from the same patient (green) and primary-primary biopsy pairs from the same patient (blue). The orange diagonal indicates where the prediction for each biopsy in a pair is identical.

From 10 patients, we obtained relapse biopsies taken between 259 and 2098 days after the diagnostic biopsy. That made it possible to determine how the predicted sensitivity changes during a treatment course. Fig. 4 shows the predicted sensitivity to CHOP in the 10 matching primary-relapse biopsy pairs. There is not much indication of selection of CHOP resistance since there are just as many pairs above the diagonal as there are below. The correlation in predicted sensitivity (CC = 0.40) is similar for another drug not used in treatment: Treanda (bendamustine, Cephalon Inc. (CC = 0.49)). We found no correlation between the time from primary to relapse biopsy and the difference between prediction in primary and relapse biopsy.

For comparison, two pairs of primary biopsies from different lymph nodes in the same patient are shown.

### Interaction between CHOP profile and cell cycle control

**Fig 5:**
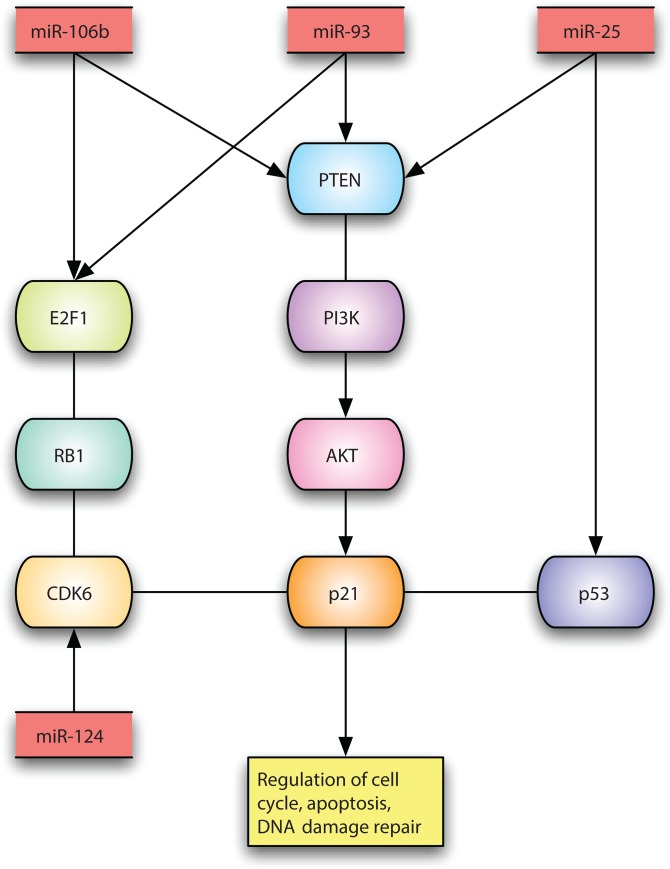
Illustration of validated interactions between microRNAs in the CHOP profile and cellular genes. The direction and sign of interactions are not shown exhaustively. It is obvious that the cellular genes affected by the microRNAs are involved in cancer signaling and drug resistance: cell cycle control, apoptosis and DNA damage repair. microRNAs are shown in red, protein-coding genes are shown in other colors.

We looked for verified interactions between the 20 microRNAs that constitute the CHOP profile and cellular genes using NCBI Gene (http://www.ncbi.nlm.nih.gov/gene). The clinically validated interactions are shown in the legend of Table 2 and Fig. 5. It has been demonstrated that miR-106b overrides a doxorubicin-induced DNA damage checkpoint [15]. miR-106b, miR-93 and miR-25 form a cluster, all expressed from the same intron. It is remarkable that out of 20 microRNAs predictive of response to CHOP, only 4 can be related to known pathways.

## Discussion

We have shown that a microRNA predictor developed based on the NCI60 panel is able to predict the response to treatment in DLBCL patients. This is despite the fact that only one B-cell line is present in the NCI60 panel. We conclude that the sensitivity or resistance of cell lines to drugs mainly depends on the presence of mutations that also determine sensitivity or resistance to drugs in patients. The mutations affect the global microRNA profile.

The predictors are highly specific to the treatment. Thus, the CHOP predictor is less accurate at predicting response in patients treated with CHOEP, and the CHOEP predictor is less accurate at predicting response in patients treated with CHOP (data not shown). This opens the possibility to use a drug specific predictor to select the optimal treatment in second or third line, where the probability of response is much less than in first line.

Of the five patients that died before response evaluation, two died within 56 days due to toxicity. They have a predicted low sensitivity to R-CHOP, thus influencing the statistical test of prediction accuracy. This could be misleading, as death due to toxicity is not the same as death due to progression of disease. If this category is excluded from the Pearson correlation, the p-value is still significant at 0.045.

All patients in this cohort were treated with Rituximab as well. However, we do not have NCI60 data on Rituximab, so no predictor of Rituximab has been developed. Remarkably, the prediction based only on three drugs from the R-CHOP combination is still able to predict remission with statistical significance. The accuracy of prediction is likely to improve once a Rituximab predictor has been developed. After unblinding we added a prednisolone (the active metabolite of prednisone) NCI60 vector but found that it did not improve predictions. Prednisolone shows no cytotoxic effects in the NCI60 assay, only moderate growth inhibition. Surprisingly, the prednisolone predictor alone was a very good prognosticator (hazard ratio 12 on overall survival, those predicted sensitive to prednisolone died much earlier). This unexpected finding needs confirmation in another study.

In building a predictor of combination therapy we summed the GI50 vectors for each of the drugs that is part of the combination therapy. We could also have chosen to combine the biomarkers for each of the drugs developed separately, but in retrospective analysis of mRNA based predictors in DLBCL cohorts (see Statistical Analysis Plan, S1 File) we found the former to give a better result. It is also possible that a combination of all drugs in one NCI60 assay would have given a better result, for example by modeling interactions more complicated than simple addition, but we did not have access to such data.

We tried to analyze microRNAs negatively correlated to GI50 as well, both in the present cohort and in the explorative cohort described in the statistical analysis plan S2, but they had no predictive power. One possible conclusion is that resistance is not as common as lack of expression of required pathways. Fig. 5 also suggests that development of resistance is not common, in contrast with other tumor types.

Cyclophosphamide is a therapeutically inactive prodrug that is converted to active metabolites by cytochrome P450 primarily in the liver. In fact the difference in GI_50_ between the 60 cell lines is only 2.8 fold. As a result, the contribution of the cyclophosphamide GI_50_ . profile to the 20-microRNA CHOP profile is limited, as seen in Table 2. When we tried to build a cyclophosphamide predictor based on NCI60 data of the active metabolite phosphoramide mustard cyclohexylamine salt, it performed much worse than the cyclophosphamide prodrug predictor. These tests were all performed after unblinding of the clinical data, as the predictor was finalized before unblinding.

We estimated tumor cell content in an adjacent FFPE slice. It ranged from 5% to 90%. This allowed us to estimate whether tumor cell content had any effect on prediction. In fact the effect size (ratio between predicted sensitivity in responders and non-responders) was similar in patients with low tumor cell content (5–20%, n = 13) and high tumor cell content (70–90%, n = 83). For patients with low tumor cell content the effect size was 1.14 (0.81–1.44). For patients with high tumor cell content the effect size was 1.15 (0.97–1.29). Thus it is not possible to conclude that lower tumor cell content affects prediction accuracy negatively.

There is only one overlap between the microRNA profile reported here for sensitivity to CHOP and previously reported microRNA profiles prognostic of survival in DLBCL patients treated with R-CHOP [5, 6]. That is miR-93. miR-93 is part of the miR-106b-25 cluster, where all members are part of the CHOP microRNA profile. This cluster resides within intron 13 of the gene MCM7 and has previously been identified as proto-oncogenic via targeting of PTEN mRNA [16]. Both miR-106b and miR-93 have also been shown to target E2F1 effectively inhibiting its translation [17]. Consistent with our observation that overexpression of miR-106b and miR-93 predict sensitivity to CHOP, E2F1 expression has previously been associated with poor survival of breast cancer patients treated with FEC [18]. FEC and CHOP share the anticancer drug cyclophosphamide.

It has been demonstrated that microRNAs form a network [19]. Thus, it would make sense to analyze microRNA data as a network. After unblinding of the clinical study we filtered the CHOP response predictor through a microRNA network as described previously for mRNA networks [20]. This improved the predictive performance, but needs to be verified in an independent test set.

It is remarkable that the predicted sensitivity to CHOP differs comparatively little between primary biopsies and relapse biopsies. There is no evidence of selection of CHOP resistance, as there are just as many biopsy pairs above the diagonal as below the diagonal in Fig. 4. This means that the diagnostic biopsy could also be useful for predicting second line therapy in relapse patients as well. This was demonstrated for relapse patients, where the diagnostic biopsy was about as good as the relapse biopsy at predicting response.

In conclusion, we have developed predictive miRNA profiles for DLBCL patients that can identify patients that will respond poorly to treatment with CHOP. The potential clinical utility lies in second and third line treatment, however, where the probability of response is smaller, and the number of available treatment options is large. Our results show that there is a potential that the predictor can assist in the selection of the optimal treatment.

## Supporting Information

S1 File Statistical Analysis Plan. (DOCX)

S1 Table Lists of microRNAs for all drug combination predictors used. (DOCX)
