# Supplementary material for: Development and Blind Clinical Validation of a MicroRNA Based Predictor of Response to Treatment with R-CHO(E)P in DLBCL
Source: PLoS One. 2015 Feb 18;10(2):e0115538. doi: 10.1371/journal.pone.0115538 (PMC4333339; doi:10.1371/journal.pone.0115538)
Supplement: S1 File — (DOCX) [file pone.0115538.s001.docx]

**Statistical Analysis Plan**

For Collaboration between Rigshospitalet and Medical Prognosis Institute on a microRNA FFPE-based predictor of response to R-CHOP

*Version 1.0 October 24, 2011*

**Background and Purpose**

MPI has developed response predictors for chemotherapy drugs tested in NCI60 by correlating differences in sensitivity to a drug to differences in microRNA expression in the cell line. Response predictors for the drugs cyclophosphamide, vincristine and adriamycin have been combined into a response predictor for CHOP. A predictor for Rituximab is not yet available due to the lack of NCI60 Rituximab data.

**Figure 1.** Logistic regression showing correlation between predicted sensitivity and clinical outcome (survival). Based on mRNA expression data from 414 DLBCL patients treated with (R)-CHOP from Lenz et al (2008) . All scores have been normalized to scale from 0 to 100 for comparison.

This approach has been tested on mRNA extracted from fresh biopsies from 414 DLBCL patients (Lenz, 2008). Figure 1 shows that the mRNA-based response Prediction score (0-100) is not better than the already available International Prognostic Index (IPI, based on clinical covariates) at predicting probability of survival after (R)-CHOP treatment. But if the two are combined, Prediction - 25*IPI, the combination performs better than either of the individual scores. This is the true promise of the mRNA-based predictor. Figure 2 shows the same data but after applying a cutoff to the prediction scores to divide patients into sensitive and resistant. Again, the combination of mRNA based Prediction and IPI is superior to either score alone.

**Figure 2**. Area under the curve (AUC) comparisons in a Receiver Operating Characteristic for the prediction versus clinical outcome. The higher the AUC, the better sensitivity and specificity.

The mRNA based predictor is of limited practical use because of its requirement for fresh biopsy tissue. Thus what we want to test in the present analysis is the ability of a new FFPE microRNA based prediction score to perform better than the existing IPI.

**Power calculations**

The predictors are fully developed using in vitro data, but the optimal cutoff separating responders from nonresponders may need refinement from clinical data. Blind testing requires the cutoff to be specified before analysis. For that reason, a statistical analysis that does not require a cutoff -- comparing the prediction scores of clinical responders and nonresponders – is preferred (one sided Wilcoxon test). Power calculations for this test based on the mRNA results shown above show that we need at least 23 nonresponders and 23 responders to get one-sided significance in the Wilcoxon test for the combined Predictor and IPI score. This will be the primary analysis. For the predictor alone, 66 non-responders would be required.

**Analysis plan**

1. MPI will use its NCI60-derived CHOP predictor to make blind predictions on the sensitivity of 144 DLBCL patients based on diagnostic FFPE samples with reasonable tumor cell content received from Rigshospitalet. Of these, 37 have died, meeting the power calculation for the combined prediction and IPI score. It is expected that 10-20 of the 144 patients will not yield sufficient microRNA for analysis. Some patients have been treated with R-CHOEP.
2. MPI will send the blind predictions of sensitivity to CHOP and CHOEP to Rigshospitalet by email. Both are on a scale from 0 to 100 and can be pooled in a statistical analysis.
3. Then Rigshospitalet will send MPI clinical outcome data, treatment information, as well as IPI and other clinical covariates where available. The data will not be traceable to any individual patient.
4. MPI (and statistician at Rigshospitalet) will combine the blind prediction scores with IPI according to the following formula:
   - 1. Combined score = prediction - 25 * IPI
5. *Primary analysis*: the Combined scores for responders and nonresponders will be compared in a one-sided Wilcoxon test. If the one-sided p-value is less than 0.05 we will reject the null hypothesis that the combined scores are equal in the two groups or higher in the nonresponders than in the responders.
6. A logistic regression and ROC plot (as shown in Figures 1 and 2) will be performed
7. For comparison, analysis 5-6 will be repeated for the Prediction score alone and for the IPI score alone.
8. The analysis 5-7 will be repeated with survival as clinical outcome.

**Follow-up analysis**

If the analysis in item 5 above is positive, the parties will discuss analysis of second line treatment. If Rigshospitalet is able to find information on what second-line treatment was used, and the outcome of this treatment, MPI will send blind predictions for the second line treatments to Rigshospitalet. These will be analyzed in the same manner as item 3-8 above.

**References**

G. Lenz et al. Stromal Gene Signatures in Large-B-Cell Lymphomas. *N Engl J Med* 2008; 359:2313-23
