# Supplementary material for: Development and Blind Clinical Validation of a MicroRNA Based Predictor of Response to Treatment with R-CHO(E)P in DLBCL
Source: PLoS One. 2015 Feb 18;10(2):e0115538. doi: 10.1371/journal.pone.0115538 (PMC4333339; doi:10.1371/journal.pone.0115538)
Supplement: S1 Table — (DOCX) [file pone.0115538.s002.docx]

**Table S1. Lists of microRNAs for all drug and combination predictors used.**

| **Treatment** | **List of microRNAs used to predict response** |
| --- | --- |
| CVP | hsa-miR-106b-star_st hsa-miR-1181_st hsa-miR-25-star_st hsa-miR-432_st hsa-miR-551b-star_st hsa-miR-652_st hsa-miR-671-5p_st hsa-miR-93-star_st |
| COPE | ACA48_x_st ENSG00000202498_x_st HBII-85-26_st HBII-85-6_x_st hsa-miR-106b-star_st hsa-miR-1181_st hsa-miR-124_st hsa-miR-1281_st hsa-miR-1299_st hsa-miR-140-3p_st hsa-miR-195-star_st hsa-miR-25-star_st hsa-miR-33b-star_st hsa-miR-34b_st hsa-miR-409-3p_st hsa-miR-432_st hsa-miR-551b-star_st hsa-miR-631_st hsa-miR-671-5p_st hsa-miR-766_st hsa-miR-93-star_st |
| MBVP | ACA10_s_st ACA11_st ACA13_st ACA18_x_st ACA21_st ACA40_x_st ACA41_x_st ACA48_st ACA48_x_st ACA51_x_st ACA57_st ACA61_st ACA7_s_st ACA9_st ENSG00000199411_s_st ENSG00000200879_st ENSG00000200932_st ENSG00000201859_x_st ENSG00000202252_st ENSG00000207002_st ENSG00000207002_x_st HBII-115_st HBII-135_x_st HBII-180A_x_st HBII-180C_x_st HBII-202_st HBII-239_st HBII-336_st HBII-429_st HBII-55_st HBII-85-26_st HBII-85-6_x_st U104_st U13_st U17a_st U17a_x_st U17b_st U17b_x_st U22_st U25_st U27_st U29_st U3-2_s_st U30_st U31_x_st U32A_x_st U33_st U34_st U38A_st U38B_st U41_st U48_st U49A_st U49A_x_st U49B_s_st U52_st U55_st U55_x_st U56_st U56_x_st U57_st U64_st U67_st U67_x_st U68_st U68_x_st U71a_st U71b_x_st U71d_st U71d_x_st U74_x_st U78_s_st U78_x_st U83B_st U89_st U8_x_st U95_st hsa-miR-106a_st hsa-miR-106b-star_st hsa-miR-1183_st hsa-miR-1246_st hsa-miR-124_st hsa-miR-1254_st hsa-miR-1275_st hsa-miR-1281_st hsa-miR-1299_st hsa-miR-140-3p_st hsa-miR-142-3p_st hsa-miR-142-5p_st hsa-miR-153_st hsa-miR-17-star_st hsa-miR-17_st hsa-miR-181a-star_st hsa-miR-18a-star_st hsa-miR-18a_st hsa-miR-195-star_st hsa-miR-19a_st hsa-miR-19b_st hsa-miR-20a_st hsa-miR-223_st hsa-miR-25-star_st hsa-miR-297_st hsa-miR-33b-star_st hsa-miR-34b_st hsa-miR-423-3p_st hsa-miR-423-5p_st hsa-miR-491-3p_st hsa-miR-595_st hsa-miR-631_st hsa-miR-663b_st hsa-miR-671-5p_st hsa-miR-766_st hsa-miR-768-3p_st hsa-miR-768-5p_st hsa-miR-769-5p_st hsa-miR-874_st hsa-miR-877-star_st hsa-miR-92a-1-star_st hsa-miR-92a_st hsa-miR-93-star_st |
| MTX | ACA10_s_st ACA18_x_st ACA48_x_st ACA51_x_st ENSG00000199411_s_st ENSG00000200879_st HBII-180A_x_st HBII-180C_x_st HBII-202_st HBII-429_st HBII-55_st U104_st U17a_st U17b_st U17b_x_st U25_st U26_st U27_st U29_st U3-2_s_st U30_st U31_x_st U33_st U38A_st U48_st U49A_st U49A_x_st U49B_s_st U49B_x_st U55_st U55_x_st U56_st U56_x_st U57_st U67_st U67_x_st U74_x_st U78_x_st U89_st hsa-miR-106a_st hsa-miR-1246_st hsa-miR-1254_st hsa-miR-1275_st hsa-miR-17-star_st hsa-miR-17_st hsa-miR-18a-star_st hsa-miR-18a_st hsa-miR-18b_st hsa-miR-19a_st hsa-miR-19b_st hsa-miR-20a_st hsa-miR-25-star_st hsa-miR-297_st hsa-miR-33b-star_st hsa-miR-34b_st hsa-miR-663b_st hsa-miR-766_st hsa-miR-768-5p_st hsa-miR-92a-1-star_st hsa-miR-92a_st hsa-miR-936_st |
| DHAP | ACA7_s_st ENSG00000199282_st ENSG00000200879_st ENSG00000201299_x_st ENSG00000201859_x_st ENSG00000202252_st ENSG00000202498_x_st HBII-202_st HBII-336_st HBII-429_st HBII-438A_s_st HBII-55_st HBII-85-11_st HBII-85-26_st HBII-85-29_x_st HBII-85-2_x_st HBII-85-6_x_st U104_st U13_st U17a_st U17a_x_st U17b_st U17b_x_st U3-2_s_st U30_st U33_st U41_st U48_st U49A_st U49A_x_st U49B_s_st U52_st U55_st U55_x_st U57_st U67_st U71b_x_st U78_s_st U83_st hsa-miR-106b-star_st hsa-miR-1183_st hsa-miR-1207-5p_st hsa-miR-1268_st hsa-miR-1281_st hsa-miR-140-3p_st hsa-miR-150_st hsa-miR-155_st hsa-miR-181a-star_st hsa-miR-181a_st hsa-miR-181b_st hsa-miR-181c_st hsa-miR-195-star_st hsa-miR-198_st hsa-miR-20b-star_st hsa-miR-223_st hsa-miR-297_st hsa-miR-33b-star_st hsa-miR-342-3p_st hsa-miR-342-5p_st hsa-miR-34b_st hsa-miR-424-star_st hsa-miR-432_st hsa-miR-503_st hsa-miR-574-5p_st hsa-miR-613_st hsa-miR-615-3p_st hsa-miR-631_st hsa-miR-766_st hsa-miR-768-5p_st hsa-miR-877-star_st hsa-miR-92a-2-star_st hsa-miR-938_st |
| Prednisolone | ACA18_x_st ACA24_s_st ACA24_x_st ACA25_x_st ACA3-2_st ACA3_st ACA40_x_st ACA47_st ACA48_st ACA52_st ACA54_st ACA62_st ACA7_s_st ENSG00000199411_s_st ENSG00000200394_st ENSG00000200879_st ENSG00000207002_st HBII-180A_x_st HBII-202_st HBII-239_st HBII-382_s_st HBII-419_st HBII-429_st HBII-52-32_x_st HBII-55_st U104_st U17a_st U17b_st U17b_x_st U25_st U26_st U27_st U28_st U29_st U3-2_s_st U30_st U31_st U31_x_st U33_st U35A_st U36A_x_st U38A_st U38B_st U41_st U46_st U46_x_st U49A_st U50B_st U50B_x_st U50_st U55_st U56_st U56_x_st U57_st U67_st U67_x_st U68_st U68_x_st U70_x_st U73a_st U78_s_st U78_x_st U83_st U93_st U95_st hsa-miR-1181_st hsa-miR-1207-5p_st hsa-miR-1246_st hsa-miR-1268_st hsa-miR-1275_st hsa-miR-17-star_st hsa-miR-17_st hsa-miR-195-star_st hsa-miR-19b_st hsa-miR-297_st hsa-miR-571_st hsa-miR-766_st hsa-miR-768-3p_st hsa-miR-768-5p_st hsa-miR-877-star_st hsa-miR-92a-1-star_st hsa-miR-92a-2-star_st hsa-miR-92a_st hsa-miR-938_st hsa-miR-939_st |
| Cyclophosp | ENSG00000200879_st ENSG00000202252_st HBII-142_st HBII-142_x_st HBII-202_st U42A_st U59B_st hsa-miR-1202_st hsa-miR-184_st hsa-miR-187_st hsa-miR-191_st hsa-miR-196a_st hsa-miR-203_st hsa-miR-29b-2-star_st hsa-miR-29c-star_st hsa-miR-328_st hsa-miR-375_st hsa-miR-423-3p_st hsa-miR-423-5p_st hsa-miR-449a_st hsa-miR-449b_st hsa-miR-489_st hsa-miR-768-3p_st |
| Doxorubicin | ACA13_st ACA48_x_st U104_st U55_st U55_x_st U74_x_st hsa-miR-106a-star_st hsa-miR-106b-star_st hsa-miR-106b_st hsa-miR-124_st hsa-miR-1281_st hsa-miR-1299_st hsa-miR-140-3p_st hsa-miR-195-star_st hsa-miR-297_st hsa-miR-29b-2-star_st hsa-miR-33b-star_st hsa-miR-342-3p_st hsa-miR-342-5p_st hsa-miR-34b_st hsa-miR-432_st hsa-miR-550-star_st hsa-miR-629-star_st hsa-miR-629_st hsa-miR-652_st hsa-miR-654-3p_st hsa-miR-671-5p_st hsa-miR-766_st hsa-miR-768-3p_st hsa-miR-877-star_st hsa-miR-93-star_st hsa-miR-93_st |
| Vincristine | hsa-miR-106b-star_st hsa-miR-25-star_st hsa-miR-432_st hsa-miR-551b-star_st hsa-miR-671-5p_st hsa-miR-93-star_st |
| GCB/ABC 10 | hsa-miR-28-5p_st hsa-miR-138_st hsa-miR-151-3p_st hsa-miR-151-5p_st hsa-miR-182_st hsa-miR-135a_st hsa-miR-155_st hsa-miR-196a_st hsa-miR-501-3p_st hsa-miR-656_st |
| GCB/ABC 8 | hsa-miR-331-5p_st hsa-miR-151-5p_st hsa-miR-28-5p_st hsa-miR-454-star_st hsa-miR-222_st hsa-miR-144_st hsa-miR-451_st hsa-miR-221_st |

CVP=cyclophosphamide, vincristine; COPE=cyclophosphamide, vincristine, etoposide; MBVP=methotrexate, carmustine, teniposide; MTX= methotrexate; DHAP=dexamethasone, ara-C, cisplatin. Prednisone or methylprednisolone was not included in any predictor.
